# Fluctuations in food and fluid intake during a 24-h World Championship: analysis of the deviation from nutritional programs

**DOI:** 10.1080/15502783.2022.2046443

**Published:** 2022-03-30

**Authors:** Keyne Charlot, Chloé Lavoué, Julien Siracusa, Emeric Chalchat, Pierre Hertert, Cyprien Bourrilhon

**Affiliations:** a Département Environnements Opérationnels, Institut de Recherche Biomédicale des Armées, Unité de Physiologie de l’Exercice et des Activités en Conditions Extrêmes, Bretigny-Sur-Orge, France; b LBEPS, Univ Evry, IRBA, Université Paris Saclay, Evry, France; c School of Psychology, Appetite Control Energy Balance Group, University of Leeds, Leeds UK; d Research Center in Human Nutrition, Laboratory AME2P, Université Clermont Auvergne, Clermont-Ferrand, France; e Fédération Française d’Athlétisme, Paris Cedex, France

**Keywords:** Ultramarathon, real-time, nutrition, hydration

## Abstract

**Background:**

A food and fluid intake program is essential for ultraendurance athletes to maximize performance and avoid possible gastrointestinal symptoms (GIS). However, the ability to follow such a program during a race has been under-assessed. We thus investigated the fluctuations of food and fluid intake during the 24-h run World Championship of 12 elite athletes (6 men and 6 women; age: 46 ± 7 years, height: 170 ± 9 cm, weight: 61.1 ± 9.6 kg, total distance run: 193–272 km) and assessed their ability to follow their nutritional program.

**Methods:**

Real-time overall intake (fluids, energy, and macronutrients) was recorded and compared to that of their program. The temporal difference in absolute values and the degree of divergence from their program were assessed, divided into four 6-h periods. GIS were recorded during the race. A questionnaire identifying the details of their nutritional program and the self-assessed causes of their inability to follow it was completed by the participants the day after the race.

**Results:**

Water, total fluid, carbohydrates (CHO), and energy intake decreased during the last quarter of the 24-h ultramarathon relative to the first half (*p =* 0.024, 0.022, 0.009, and 0.042). However, the differences were no longer significant after these values were normalized by the number of passages in front of the supply tent. The participants progressively failed to follow their nutritional program, with the intake of their planned items dropping to approximately 50% during the last quarter. However, this was adequately compensated by increases in unplanned foods allowing them to match their expected targets. GIS, lack of appeal of the planned items, and attractivity of unplanned items were the main explanations given for their deviation from the program (64, 27, and 27%, respectively).

**Conclusion:**

Despite evident difficulty in following their nutritional programs (mostly attributed to GIS), elite ultraendurance runners managed to maintain high rates of fluid and food intake during a 24-h ultramarathon and therefore still met their planned elevated nutritional objectives.

**Abbreviations:** CHO: carbohydrates, GIS: gastrointestinal symptoms

## 1. Background

Ultramarathoners (engaged in races longer than 42.2 km) are strongly encouraged to follow food [1] and fluid [2] intake recommendations [3] to properly replace most of the vast losses induced by fuel utilization of the body and sweating/evaporation (completed by respiration and urination), respectively.

The attainment of such an objective may be made more difficult by the in-race repetitive mechanical disruption to the gut, inducing gastrointestinal symptoms (GIS) [4,5], which also may be amplified by such significant intake [6]. Observational studies have reported actual intake to be lower than that advised [7–10], lower for nonfinishers than finishers [4], and lower for slower than faster runners [7]. These results not only highlight the overall difficulty of ultramarathoners to follow the recommendations but also underline the apparent ability of elite athletes to adopt a race diet closer to recommendations, likely through a more adequate nutritional program and/or one that results in improved gut tolerance [11,12]. Other explanations roughly associated with GIS are possible (alterations of the acceptability of available foods and fluids) but have only thus far been anecdotally investigated [13,14].

An individualized approach has been advised to enhance the ability to follow nutritional recommendations by identifying the highest tolerable rates of energy and carbohydrate (CHO) intake during training sessions [1]. A nutritional program can then be designed, based on such personal empirical evidence, in which the volume and frequency of intake are *a priori* defined [15]. However, the efficiency of this anticipatory method and the degree of convergence between expected and actual intake have been insufficiently assessed [16].

To date, the vast majority of studies to record food and fluid intake have only considered total amounts rather than a real-time approach, an observation that can be easily explained by the obvious logistical difficulties in recording all intake during long races, especially those involving a one-way route. However, when adopted, such a temporal approach has allowed the identification of fluctuations in energy, macronutrient, and/or fluid intake during ultraendurance races [13,14,17,18]. Moreover, this temporal analysis may help in assessing the pertinence of designing and following a nutritional program by highlighting the periods of divergence between expected and actual intake and observing the potential effects of undesirable in-course events, such as the occurrence of GIS.

The modifications of food and fluid intake during the 24-h run World Championships of 12 members of the French national team were investigated. These intake fluctuations were considered not only by measuring absolute values but also in terms of the ability of the athletes to follow their nutritional program. The second aim was to explain potential modifications through the real-time recording of symptoms as well as by subjective post-race questionnaires. Contrary to most races, which use a one-way route race, 24-h races are designed to allow easy and very frequent access to food and fluids (laps of 1.5 km in this race) on a flat circuit under nonextreme environmental conditions (10–25°C without rain or wind). Therefore, participants were placed in more comfortable conditions than in other ultraendurance events. Indeed, they could rely on predictability of food and fluid supplies facilitating the following of their program.

## 2. Methods

### Event

This study was conducted during the 24-h ultramarathon World Championship held in Albi (France) from October 26-27, 2019. The race consisted of running the greatest distance possible over 24 h (start of the race at 10:00 am the first day). Participants ran on a short loop (1.491 km) that combined asphalt (~75%) and tartan track (~25%). Ambient conditions (dry temperature, hygrometry, and wet-bulb globe temperature (WBGT)) were continuously measured and presented in mean 6-h periods using a weather station (Kestrel Meter 5400 Heat Stress Meter, Birmingham, MI, USA) near the track at a height of 1.2 m and exposed directly to the sun.

#### Subjects

Twelve French elite athletes (six men and six women) agreed to participate in this study (age: 46 ± 7 y, height: 170 ± 9 cm, body mass: 61.1 ± 9.6 kg, body fat mass: 13.5 ± 6.5%). Their individual characteristics are presented in Figure 1. The study was conducted in accordance with the Declaration of Helsinki and was approved by the regional ethics committee (CPP Ile-de-France 8, France, registration number: 2019-A02445-52, Etude LemuR). The participants’ written informed consent was obtained after they were informed of the purpose and procedures of the study.

**Figure 1.**
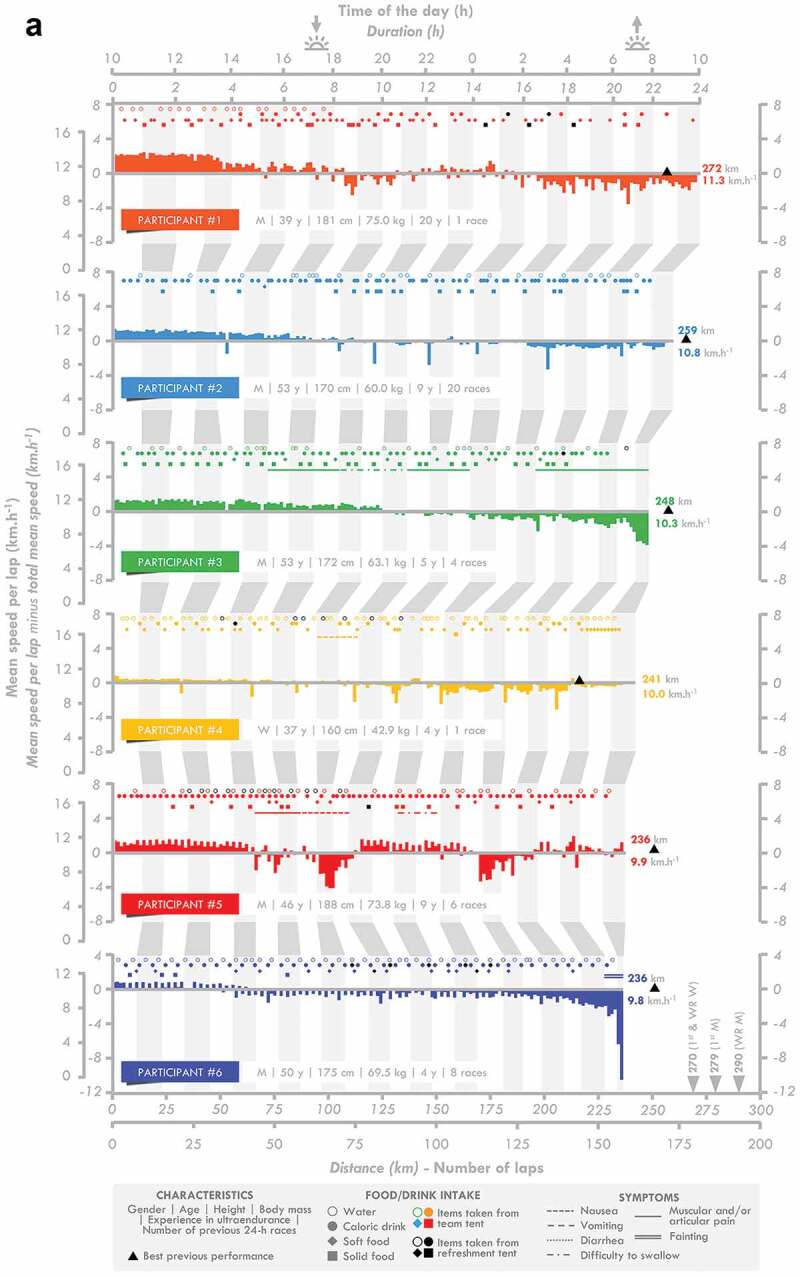
**Individual race individual**. M: man, W: woman, WR: world record.

**Figure 1.**
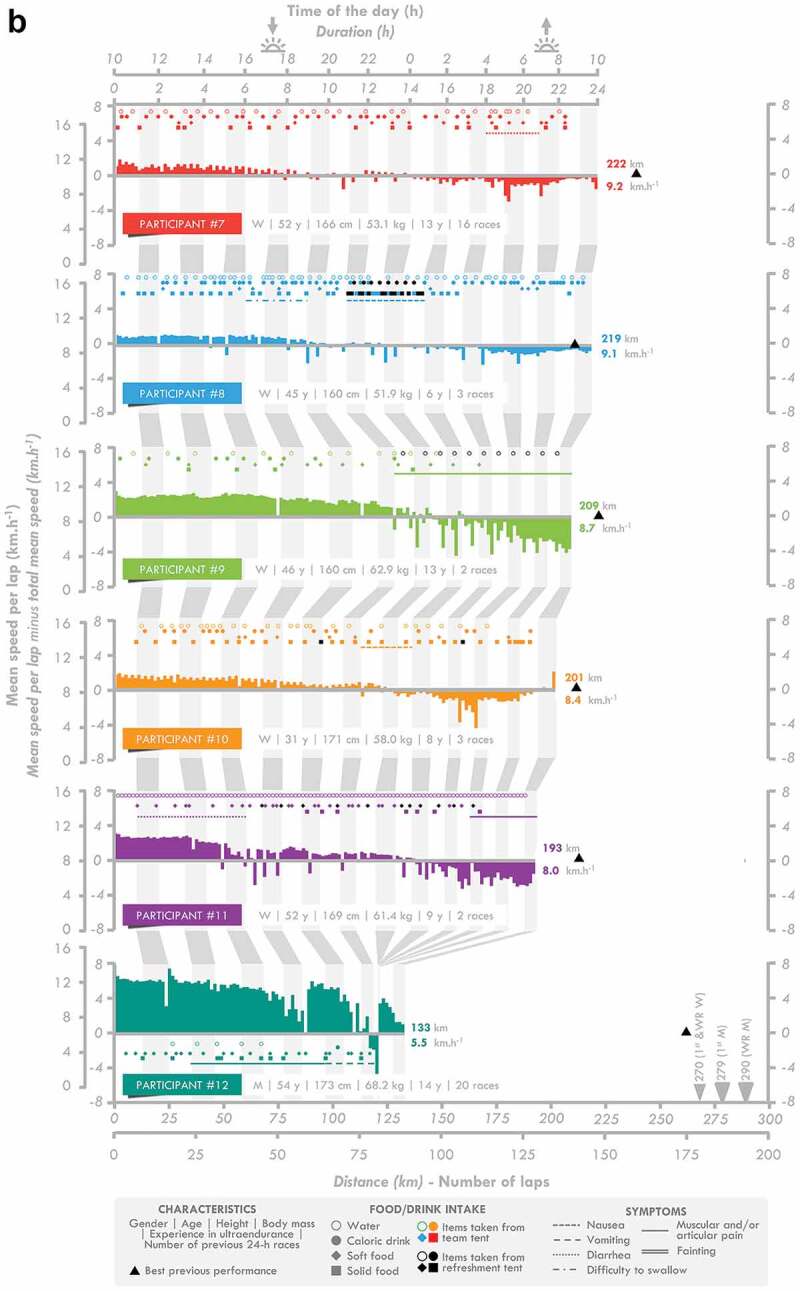
(Continued).

#### Design

Prior to the race, each participant provided their nutritional program. They indicated at which lap they wanted to consume a specific drink and/or food with the corresponding quantity/volume during their passage in front of the France team tent. The nutritional composition of every food and fluid (even those that were not included in the program and those that were brought by managers for collective use) was collected in a spreadsheet. Four participants who consumed home-made food (less than 10 items in total) were asked to provide their recipes to establish the composition. Food and/or drinks were handed out according to the individual programs of the athletes. Even if no intake was programmed, food and drinks from their selection were still available on a tray to allow the participants to pick one if necessary. Participants were then free to modify their program and ask for unplanned or common foods. Indeed, a selection of food and drinks was available in large amounts for all participants. All consumed food and drinks were registered, along with the amount consumed (in g or ml), using an individual chart displaying the programmed items and quantities consumed during the run. When an item was consumed according to the program (i.e. consumed on the intended lap), it was circled. If the quantity differed, it was corrected using a blank column. Finally, the consumption of unplanned items was recorded, along with their quantity, in the same blank columns. The participants were separated into three groups of four, and each group was assisted by two members of the staff with their nutritional and logistical support throughout the race.

A refreshment tent provided a complementary source of food and drinks, providing mostly water, cakes, fruit, and mashed potatoes. Participants were asked to indicate the amount consumed after the race (less than 2 h later). They were then asked once again 24 h later to check that the answers were concordant.

#### Methodology of data collection

##### Food and fluid intake

The hourly food and fluid intake was calculated for each participant using spreadsheets (Excel for Office 365, Microsoft, Redmond, WA, USA). The origin of the food (solid and soft foods, i.e. those that did not require chewing) and fluid (water and caloric fluids, i.e. with energy content) intake and energy, macronutrient (carbohydrate, fat, and protein), and micronutrient intake (caffeine and sodium) were assessed. Table 1 presents the different items consumed during the race corresponding to each category of foods and fluids.

**Table 1.** List of foods and fluids consumed by participants

Solid Food	Solid Food	Caloric Fluids
Fruits [10]	Biscuit [1]	Soda [9]
Cereal bar [7]	Chocolate [1]	High-CHO sport drink [5]
Protein bar [5]	Potato [1]	Chicken broth [5]
Cold meat [3]	Marzipan [1]	Fruit juice [5]
Sweet potatoes [3]	Rice [1]	High-PRO sport drink [2]
Cheese [3]	Bread [1]	Energy drink [1]
Nuts [3]	Cheese sandwich [1]	
Rice pudding [2]	Soft Food	Non caloric fluids
Cracker [2]	Stewed apples [8]	Water [11]
Fruit bar [2]	Mashed potatoes [4]	Sparkling water [8]
Gingerbread [2]	Energy gel [4]	Coffee [6]
Cake [2]	High-protein cream [2]	Tea [1]
Savory cake [1]	Honey [1]	

The number of participants having consumed an item is indicated in brackets

Then, the absolute intake (in mg, g, kg, mL, L, kJ, or MJ per hour) of the various categories was calculated. The number of times the participants consumed food and/or drinks per hour (i.e. instances of consumption [17]) was counted. The frequency of intake (instances of consumption divided by the number of laps per hour) was then deducted. This variable reflects the choices made by the participants to select and therefore consume foods/drinks despite a similar level of availability. A reduction in the frequency of intake of a specific group of foods/drinks implied a choice made by the participants to consume less of it. Indeed, it has been clearly observed that participants of 24-h ultramarathons are unable to maintain the same running pace, regardless of their level of performance [19]. It was therefore necessary to normalize their intake by the frequency of the possibility to resupply. Their “relative intake” was calculated by dividing their absolute intake by the number of laps per hour to also take into account the very likely decrease in the frequency of the possibility to feed and/or drink.

Their actual intake was also compared to that which was expected (based on the program). The hourly degree of convergence (actual/expected intake × 100) for energy, CHO, and fluid intake (the three most important categories of intake contained in multiple benchmark recommendations [3]) was calculated for the entire race. First, only the items included in their program were considered in the calculation of actual intake to assess their ability to conform to their nutritional program. In a second analysis, all items were considered to assess their ability to conform to their expected intake by consuming unplanned items.

#### Symptomology

Each hour, participants were asked by the team physician whether they experienced GIS and/or other symptoms (e.g. articular or muscular pain). Before the race, they were given a list of GIS they could experience (difficulty swallowing, belching, acid reflux, heartburn, nausea, vomiting, abdominal pain, bloating, flatulence, urge to defecate, diarrhea, and constipation) to facilitate their identification during the race. Occurrences of GIS were therefore noted in real-time. These observations were cross-checked with the athletes a few hours after the race. Thus, the duration of the symptoms but not their intensity was recorded, as the participants were not comfortable with rating these symptoms during the race and the accuracy of their post-race recollection was not reliable.

#### Questionnaires

Before the race, the participants were asked to indicate with whom they usually design their nutritional program. Then, their knowledge of sports nutrition and the importance of their nutritional program on their performance were assessed on a 100-mm horizontal visual analog scale preceded by the following question: “According to you, what is your level of knowledge in sports nutrition?” and “Does your nutritional program have an influence on your performance?” The scale was anchored by “Very low” and “Very high” and “Not at all” and “Extremely” at the left and right ends, respectively. The distance from the extreme left to the participant’s vertical dash represented the rating score, expressed in mm. The two following questions were then asked: “Did you manage to follow your program during your previous 24-h ultramarathon(s)?” and “At what moment during your previous 24-h ultramarathon(s) did you ceased to follow your program?”, with “Absolutely Not” and “Entirely” and ”0 h” and “24 h” at the left and right ends, respectively. Finally, participants had to answer the following question “For which reason(s) did you cease to follow your program?” by selecting none, one, or several preselected or free answers.

The morning after the race, the last three questions were asked again using the last 24-h race as the reference: “Did you manage to follow your program during this 24-h ultramarathon?”, “At what moment of this race did you cease to follow your program?”, and “For which reason(s) did you cease to follow your program?”

#### Statistical analysis

Statistical analyses were performed to assess the temporal fluctuations in instances of consumption, frequency of intake, absolute and relative intake (divided by the number of laps completed), and the degree of divergence between the actual and planned intake. The race was therefore separated into four 6-h periods (0–6 h, 6–12 h, 12–18 h, and 18–24 h). The data were not normally distributed according to Shapiro–Wilk tests. Friedman’s tests were therefore performed, with a post hoc analysis by Wilcoxon signed-rank tests. One participant (#12) abandoned the race due to muscle pain. He was thus excluded from the analyses with the exception of the pre-race questionnaires. Data are presented as the means ± SD. Significance was defined as *p <* 0.05. Analyses were performed using Jamovi software (1.2.9 version, the Jamovi project; retrieved from https://www.jamovi.org).

## 3. Results

### Race details

The speed per lap, instances of consumption, and the occurrence of gastrointestinal (see Figure 4 for an overview) and other symptoms indicated for the 24-h ultramarathon participants are presented in Figure 1. Changes in the meteorological measurements during the race are presented in Table 2. The speed and number of laps completed by the participants progressively decreased during the race (*p =* 0.001 for both). The decrease in speed occurred in two steps, with the first observed between the 0–6 and 6–12 h periods and the second between the 12–18 and 18–24 h periods.

**Table 2.** Evolution of environmental variables, pacing, instances of consumption, and intake per lap during the race

	0–6 h	6–12 h	12–18 h	18–24 h
**General outcomes**				
Temperature (°C)	22.3 [18.2 – 24.3]	19.8 [16.1 – 23.6]	14.1 [13.3 – 15.1]	13.5 [12.2 – 16.5]
Hygrometry (%)	54.3 [47.2 – 62.9]	63.0 [48.4 – 79.4]	88.1 [84.6 – 91.3]	90.6 [83.0 – 92.7]
WBGT (°C)	21.6 [18.4 – 23.1]	16.6 [14.3 – 19.7]	13.2 [12.4 – 13.7]	12.9 [11.3 – 17.3]
Speed (km.h^−1^)*	11.0 ± 0.9	10.0 ± 0.9^αα^	9.4 ± 1.1^ααα^	8.5 ± 1.5^αααβββγ^
Laps completed (n)*	44.3 ± 3.7	40.0 ± 3.8^αα^	37.3 ± 5.0^ααα^	33.5 ± 6.2^αααβββγγ^
**Instances of consumption**				
Water	12.6 ± 8.2	11.3 ± 7.1	9.3 ± 6.9	7.3 ± 5.6^αααβ^
Caloric drink	10.4 ± 7.0	10.2 ± 6.4	8.6 ± 7.0	7.4 ± 6.6^αβ^
Total fluid	23.0 ± 7.2	21.5 ± 8.2	17.9 ± 8.0^ααα^	14.6 ± 8.1^αααβββ^
Solid food	4.6 ± 3.6	4.9 ± 3.8	5.0 ± 5.5	2.4 ± 2.5
Soft food	4.2 ± 4.6	5.6 ± 4.9	4.5 ± 3.7	3.3 ± 4.6
Total food	8.8 ± 4.9	10.5 ± 5.8	9.5 ± 4.6	5.6 ± 4.5
All foods and fluid	31.8 ± 8.3	32.0 ± 10.2	27.4 ± 9.4	20.3 ± 9.0^αααβββγ^
**Frequency of intake (%)**				
Water	29 ± 21	29 ± 22	26 ± 22	24 ± 21
Caloric drink	24 ± 15	25 ± 16	22 ± 17	20 ± 18
Total fluid	53 ± 18	55 ± 23	48 ± 21	44 ± 25^β^
Solid food	11 ± 9	12 ± 9	14 ± 16	7 ± 8
Soft food	9 ± 10	14 ± 13	12 ± 11	9 ± 12
Total food	20 ± 10	27 ± 15	26 ± 14	16 ± 12
All foods and fluid	73 ± 20	81 ± 30	74 ± 28	60 ± 26^ββγ^
**Intake per lap**				
Water (mL)	60 ± 40	65 ± 39	62 ± 40	46 ± 31
Caloric drink (mL)	44 ± 41	58 ± 48	43 ± 48	46 ± 49
Total fluid (mL)	105 ± 35	124 ± 57	105 ± 52	92 ± 59
Solid food (g)	8.5 ± 6.6	9.1 ± 8.1	8.2 ± 6.3	5.0 ± 5.0
Soft food (g)	13.3 ± 20.1	17.5 ± 20.7	18.8 ± 17.4	14.7 ± 23.7
Total food (g)	21.8 ± 21.7	26.6 ± 22.5	26.9 ± 15.1	19.7 ± 22.7
Energy (kJ)	240 ± 125	244 ± 106	209 ± 77	184 ± 140
Carbohydrate (g)	10.1 ± 6.0	10.8 ± 5.3	8.9 ± 3.6	7.8 ± 4.9
Fat (g)	1.3 ± 1.1	1.1 ± 0.8	1.1 ± 1.1	1.0 ± 1.8
Protein (g)	1.3 ± 1.1	1.2 ± 1.2	1.2 ± 0.9	0.9 ± 1.1
Sodium (mg)	115 ± 77	167 ± 95	152 ± 90	99 ± 83
Caffeine (mg)	0.72 ± 0.91	1.94 ± 1.86	2.92 ± 2.77	2.27 ± 2.91

Mean [minimum–maximum] or mean ± SD. α different from the 0–6-h period, β different from the 6–12-h period, γ different from the 12–18-h period. One symbol *p* < 0.05, two symbols: *p* < 0.01, three symbols: *p* < 0.001.

*Retrieved from the organization official chronometer (https://www.breizhchrono.com/detail-de-la-course/crs_id/13092/ for men and https://www.breizhchrono.com/detail-de-la-course/crs_id/13094/ for women).

### Intake analysis

The instances of consumption of water (*p =* 0.005), caloric drinks (*p =* 0.020), total fluids (*p =* 0.010), and all food and fluids (*p =* 0.010) significantly fluctuated during the race (Table 2). Water, caloric drinks, and total fluids were less consumed during the 18–24 h than the 0–6 h (*p = *0.001, 0.019, and 0.001, respectively) and the 6–12-h periods (*p =* 0.016, 0.031, and 0.001, respectively). The instances of consumption of all food and fluids were lower during the 18−24 h than the 0–6 h, 6–12 h, and 12–18 h periods (*p =* 0.001, 0.001, and 0.038, respectively).

There was a time effect for the frequency of the total fluid (*p =* 0.029) and all fluid and food intake (*p = *0.017) (Table 2). The frequency of total fluid intake was lower during the 18–24 h than the 6–12-h period (*p =* 0.021). For total food and fluids, it was lower during the 18–24 h than the 6–12-h (*p =* 0.009) and the 12–18-h periods (*p =* 0.045).

**Figure 2.**
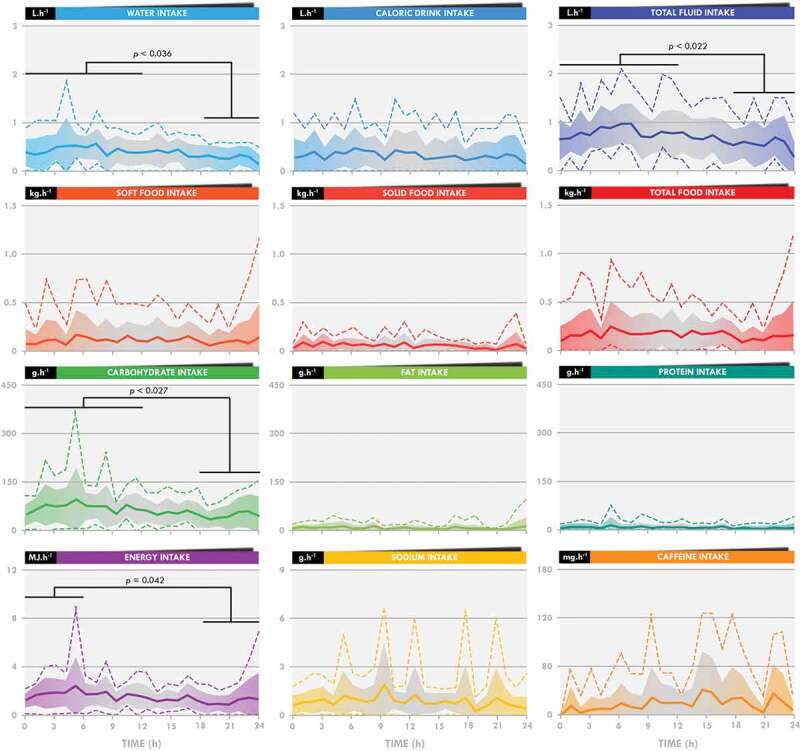
**Temporal evolution of absolute intake during the 24-h race**. Bold lines indicate the means, the filled colored bars the standard deviations, and the dotted lines the maximal and minimal values.

There was also a time effect for absolute water (*p =* 0.032), total fluid (*p =* 0.006), CHO (*p =* 0.008), and energy intake (*p =* 0.047) (Figure 2). Post hoc tests showed water, total fluid, and CHO intake to be lower during the 18–24 h than the 0–6 h (*p =* 0.024, 0.022, and 0.009, respectively) and 6–12 h periods (*p =* 0.036, 0.004, and 0.027, respectively). Energy intake was lower during the 18–24 h than the 0–6-h period (*p =* 0.042). Relative intake (absolute intake divided by the number of completed laps) was not statistically different between the different phases of the race, as there was no time effect (Table 2).

### *Expected* vs *actual intake*

**Figure 3.**
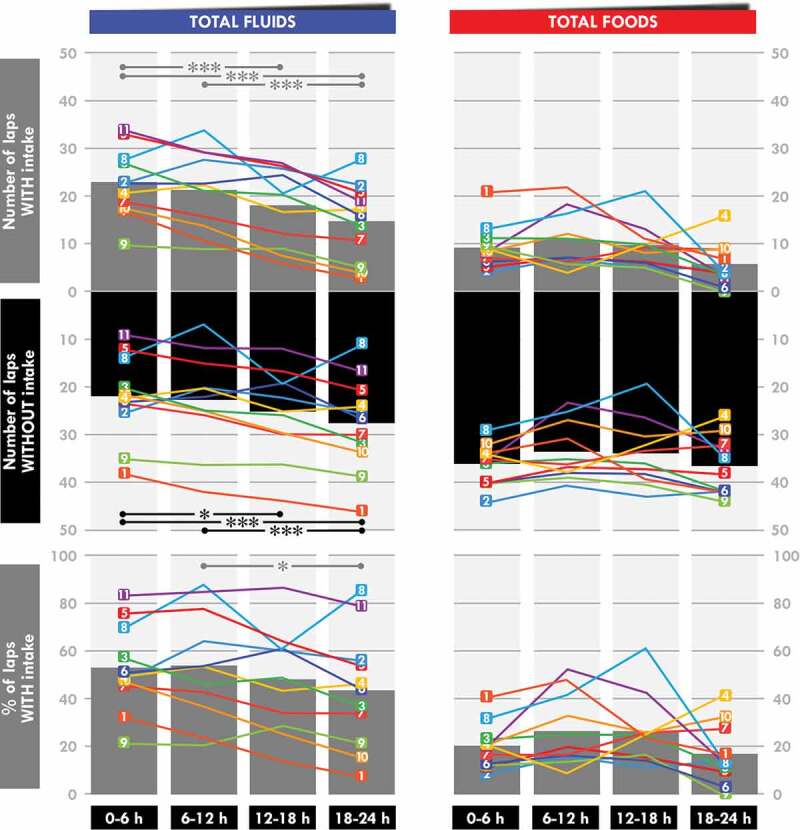
**Temporal evolution of the degree of convergence from the expected energy and total fluid intake**. Vertical bars indicate the means and the lines the individual evolution. *Significant difference between the two linked 6-h periods. One symbol: *p* < 0.05, two symbols: *p* < 0.01, three symbols: *p* < 0.001.

In the first set of analyses, only the items included in the nutritional program were considered. There was a time effect in the degree of convergence of the actual intake compared to the expected ones for three intake categories of interest: energy (*p =* 0.002), CHO (*p =* 0.002), and total fluid (*p =* 0.001) (Figure 3). For energy and CHO intake, the relative difference between the expected and actual intake was larger for the 18–24 h than the 0–6 h (*p <* 0.001 for both) and 6– 12 h (*p =* 0.027 for both) periods. It was also lower for the 12–18 h than the 0–6 h (*p =* 0.001 for both) period and for the 6–12 h than the 0–6-h period (*p =* 0.017 for both). For total fluid intake, it was higher for the 18–24 h than the 0–6 h (*p <* 0.001), 6–12 h (*p =* 0.032), and 12–18 h (*p =* 0.025) periods. It was also higher for the 6−12 h (*p =* 0.005) and 12–18 h (*p =* 0.006) periods than the 0–6-h period.

The second set of analyses, which included all items consumed, showed a time effect for fluids only (Figure 3) (*p =* 0.043).

### Questionnaires and symptomology

**Figure 4.**
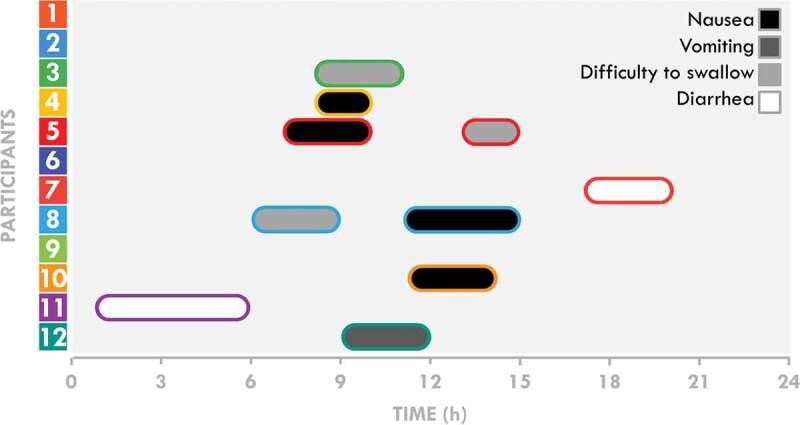
Occurrences and duration of gastrointestinal symptoms during the 24-h race.

All GIS occurred at mid-race (between 6 and 15 h), with the exception of two episodes of diarrhea (Figure 4).

**Figure 5.**
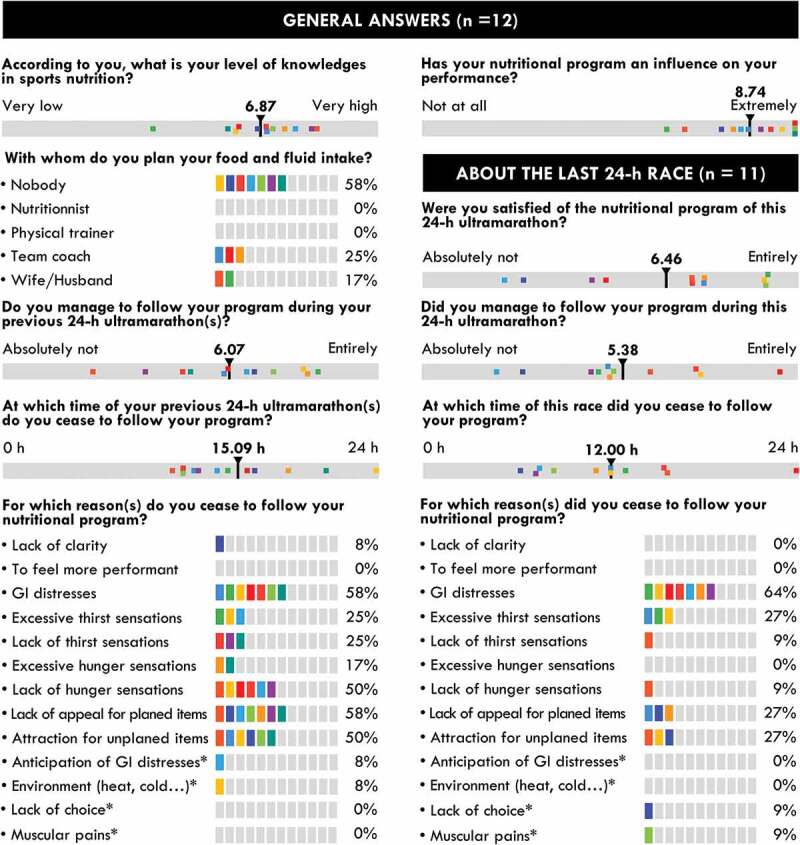
**Individual and mean responses to the pre- and post-race questionnaires**. Vertical black bars indicate the mean values and colored squares indicate individual answers.

The mean and individual responses to the pre- and post-race questionnaires are shown in Figure 5. All but one participant estimated that they possessed higher than moderate knowledge of sports nutrition and none of them sought help from nutrition professionals to design their nutritional program. All estimated that their program had a large influence on their performance.

The nutritional program was estimated to be moderately followed (in their previous races for the same length of time as in the present one) and they estimated that they were unable to adhere to their program starting from approximately mid-race, although the variability of responses was very large. GIS were the main reason given for the lack of compliance (64%). Other reasons given were a lack of appeal for their planned items or the attractivity of unplanned items (50–58% for previous races and 27% for both reasons for the present race).

## 4. Discussion

Water, total fluids, CHO, and energy intake decreased during the last quarter of the 24-h ultramarathon (18–24 h). However, these decreases were mostly explained by the reduction in the number of passages in front of the team tent as the running speed decreased and therefore by the reduction in the instances of consumption. The participants increasingly diverged from their nutritional program but quickly compensated for it with unplanned foods to match the expected fluids, CHO, and energy intake. The main explanation for not following the program was the occurrence of GIS, followed by the lack of appeal of planned items and the attractivity of unplanned items. All GIS were upper and occurred at mid-race (between 6 and 15 h), except for two cases of lower GIS (diarrheas exclusively), and certain symptoms were associated with obvious modifications of eating behavior. These results indicate that despite evident difficulties in following their nutritional programs (mostly attributed to GIS), these elite participants managed to maintain elevated levels of fluid and food intake during a 24-h ultramarathon, the decrease in absolute intake very likely being explained by the reduction in the rate of availability of their food supplies.

In this study, fluid, CHO, and energy intake were lower in the last quarter (18–24 h) of the race than in the first half (0–12 h) (Figure 2). This decrease did not prevent the participants from largely reaching the overall minimal nutritional recommendations [3]. This may result from 1) an inability to maintain and/or 2) the choice to limit intake during this crucial phase of the race. Unlike one-way route races, during which food and drink supplies are less frequent, the design of the 24-h World Championship allows passage in front of the National and refreshment tents each 1.5 km, therefore multiplying and facilitating access to supplies. As a consequence, any decrease in speed would automatically result in a decrease in the frequency of being able to eat and/or drink. The running speed significantly decreased for almost all participants (except # 4 and 5) (Figure 1 and Table 2), in accordance with previous observations [19], resulting in a decrease in the number of instances of drinking, but not eating, during the last quarter. Analysis of the frequency of intake shows that they did not compensate by increasing the rate of fluid intake when passing in front of the tent. It reveals rather that they maintain their rate of intake, although a slight decrease in the frequency of drinking was observed. Logically, intake was normalized by the number of laps completed and did not change during the race, clearly indicating that these elite participants successfully maintained their intake during the 24-h race, the observed reductions in intake being explained simply by a reduction in their running speed.

Fluctuations in food and fluid intake during ultraendurance races have only been poorly described [13,14,17,18], likely due to the difficulty of accurately recording real-time intake. CHO intake was observed to increase during the second half of 100 km (12 h 49 min for one female participant) [14] and 120-km ultramarathons (mean: 12 h 19 min for five male participants) [17]. These concordant results were observed in races with different constraints than ours. In the former, the sole participant did not have access to her individualized foods and fluid during the first checkpoints of the race and in the latter, intake was “*ad libitum,”* as the foods and drinks were carried by cyclists following each participant during the entire race. In a longer bike ultramarathon (1,230 km; 54 h for 14 male participants), fluid, CHO, and energy intake decreased during the second half of the race [13]. Finally, Berger et al. [18] reported decreases in energy and CHO intake after the first day of a seven-day treadmill running world record attempt (833.05 km for one female participant). Overall, these studies (including the present one) support a hypothetical three-step model that clearly requires a stronger body of evidence: 1) a progressive increase or at least maintenance of CHO intake during the first 12 h, 2) maintenance of all intake until 18–30 h of racing, and then 3) a decrease in all intake until the end of the race. It remains to be known how these observations may be used by participants to adjust their nutritional strategy.

Although no explanation was proposed by Wardenaar et al. for the 120-km race [17], Moran et al. [14] suggested that the increase in CHO intake during the second part of the 100-km race was induced by both the satiated state of the participant during the first hours of the race due to the pre-race breakfast and her inability to access her own items at the first checkpoints. In a study based on anecdotal reports [13], the decrease in intake during the second half of the race was attributed to 1) feelings of saturation induced by the ingestion of large amounts of food and drink, 2) feelings of sensory-specific satiety due to high amounts of CHO intake, and 3) the inability to maintain sustained frequent food and drink intake because of mental fatigue. Finally, in the study of Berger et al. [18], the authors indicated that the participant modified her diet on days 2 and 3 to privilege liquid CHO sources due to treadmill-induced motion sickness. With the exception of the aforementioned decrease in consumption opportunities, modifications in intake in this race were not observed.

The participants believed themselves to possess a moderate-to-high level of knowledge of sports nutrition and none sought professional advice to design their nutritional program, the large majority managing alone or with the help of their spouse/wife/husband. These answers are consistent with previous studies [20,21], in which the objective level of nutritional knowledge was estimated to be high, but with high variability [20], and for which personal and peer experience was the main source of influence, largely ahead of scientific advice in the design of hydric [22] and nutritional [23] programs, pre-race meals [24], or nutritional habits [20,25]. Their reluctance to seek professional help appears to partially contradict their beliefs that their nutritional program has a high impact on their performance. Indeed, Citarella et al. [21] showed a positive link between the level of objective nutritional knowledge and general dietary practices in ultraendurance athletes. Hence, even though experience appears to provide sufficient nutritional knowledge in terms of reaching the recommendations [15], seeking a professional would refine the food selection.

In this study, the participants deviated relatively quickly from their nutritional program. Analysis of the three main markers (fluid, energy, and CHO intake) showed that they were globally able to consume what they planned during the first quarter of the race (apart from two or three athletes), but that the degree of divergence between the actual and expected intake progressively and dramatically increased during the remainder of the race, until reaching approximately ~50% during the last quarter (Figure 3). Nevertheless, their targeted intake remained stable, indicating that they adequately compensated by consuming additional items (their personal items, those of the teams, and/or those of the refreshment tent). Only one study has previously compared planned and consumed intake during an ultraendurance race. McCubin et al. [16] carried out such an analysis during the seven-day Marathon des Sables held in the Sahara desert and found only a 4% deviation from the program. However, participants of this race minimized the quantity of food items to limit load carriage and were not offered alternative nutritional propositions, as in our study, making comparison with our study difficult. During the seven-day treadmill world record attempt [18], the authors mentioned that the participant was unable to follow her program (quantities were too large to consume during her breaks and modification of her diet induced by motion sickness) but they only affirm that her “initial nutrition strategy would have matched her energy expenditure more closely,” without publishing concrete data.

Our participants were well aware of their difficulties in following their programs (Figure 5) and were moderately accepting them, although the heterogeneity of the answers was high. The main reason for these difficulties was the apparition of GIS (64%). The etiology and frequency of GIS during ultraendurance races have been extensively described and appear to mainly result from physiological (reduction in splanchnic blood flow) and mechanical factors (pounding and jostling during running) [4,6,26,27], as well as the high intake, during the race (particularly hyperosmolar CHO solutions) [6]. Although there appears to be no difference in intake (fluids, CHO, or energy) between participants who experience GIS and those who do not [4,7,27,28], these elite athletes affirmed that GIS altered their planned nutritional intake. A real-time collection of GIS data showed that all GIS, with the exception of lower GIS (diarrhea), occurred at mid-race (6–15 h), coinciding with the nocturnal and colder period and therefore concomitantly with the observation of large and growing differences between actual and planned intake. Moreover, an episode of GIS was notably associated with an obvious modification of the intake strategy for certain participants: during an episode of nausea, participant #8 privileged the intake of fruits and water with fruit squash at the refreshment tent, participants #5 and 10 substantially decreased all intake, and participant #11 avoided consuming any solid foods during an episode of diarrhea. Although these associations are anecdotal, overall, these results strongly suggest that GIS significantly alter planned nutritional patterns, both acutely and potentially throughout the race. Other reasons less frequently mentioned by participants to explain their deviation from their program (excessive thirst, lack of appeal for planned items, and attractivity of unplanned items) may also contribute to such alterations. It is possible that monotony and alimentary chronobiology are involved. It is indeed plausible that the acceptability of certain items may decrease with repetition and that the acceptability of unplanned items (i.e. soup or mashed potatoes) may increase after dusk. These suggestions naturally require scientific evidence, as such effects have been observed in minimally transposable contexts [29–31]. Thus, even in races during which intake is *a priori* calculated, a non-negligible part of spontaneous choices remains, justifying the presence, if possible, of a large choice of fluids and foods to complement those included in the nutritional program. Interestingly, these results appear to indicate that these experienced elite athletes accept their inability to follow more than half of their program, reflecting that 1) it may be difficult to design a robust nutritional program that is resistant to intrinsic and/or extrinsic hazards and/or 2) the management of planned and unplanned intake of items is not a burdensome task for this population in these kinds of events.

## 5. Conclusions

In an ultraendurance race (24-h World Championship) during which overall fluid and food intake of elite athletes was largely considered to be in agreement with benchmark recommendations, modest decreases in water, total fluid, CHO, and energy intake during the last quarter of the race were observed. However, overall intake appeared to be stable throughout the race when it was normalized by the number of passages in front of the supply tent, reflecting an ability of these athletes to sustain an elevated rate of consumption. Interestingly, these participants, who granted a large importance to their nutritional program in their performance, progressively abandoned it without it resulting in a reduction in their targeted key intake (fluid, CHO, and energy). This apparent paradox is explained by their ability to select adequate items outside their program. GIS was the main reason given to explain their inability to strictly follow their program. These results, observed for a limited sample of elite athletes, show that designing a nutritional program that will be strictly followed during an ultraendurance race appears to be unfeasible and that, instead, they subtly adapt their intake during the race using additional items without detrimental consequences on the elevated rate of recommended intake. Thus, anticipating their inability to follow their program, making additional foods and fluids available seems recommendable. A two-step strategy, the first composed of planned food and fluid intake and the second offering a larger diversity of available items, would be helpful for maintaining the demanding nutritional aims of athletes participating in ultraendurance races.

## Data Availability

The datasets used and/or analyzed during the current study are available from the corresponding author upon reasonable request.
